# Approach to depression in Parkinson’s Disease. A case report

**DOI:** 10.1192/j.eurpsy.2025.1339

**Published:** 2025-08-26

**Authors:** B. Lopez Abellan, P. Abril Bohórquez, N. Linero Rios, R. Justicia González, N. Echeverría Hernández

**Affiliations:** 1 Psiquiatría, Complejo Asistencial de Ávila, Ávila, Spain

## Abstract

**Introduction:**

Parkinson’s Disease is associated with depigmentation of the substantia nigra and locus coeruleus, with specific pathophysiological alterations. It is characterized by tremor at rest, bradykinesia, postural instability and rigidity. But there are also other comorbid psychiatric disorders that accompany it, such as cognitive impairment, psychotic symptoms (hallucinations and delusions), mood disorders and sleep disorders, among others.

**Objectives:**

The main objective of this work is to review the current scientific evidence on the management of depression in Parkinson’s Disease.

**Methods:**

The case of a 75-year-old man with a neurological history and a diagnosis of depression with a poor evolution is presented. A detailed search was performed on UpToDate using the search terms “Parkinson’s Disease” and “Depression”.

**Results:**

This is a 70-year-old man with a history of Parkinson’s disease and comorbid depression with a poor evolution. A multitude of therapeutic options have been tried, such as SSRIs, SNRIs, antipsychotics, tricyclic antidepressants and an intensive psychotherapeutic approach. Despite what has been described, the expected improvement is not obtained and, given the difficulties in the treatment of his Parkinson’s Disease, an update is necessary according to the scientific evidence collected.

According to the scientific evidence consulted, among the antidepressants most studied in Parkinson’s Disease are SSRIs, SNRIs and tricyclic antidepressants. The choice of antidepressant treatment will depend on the patient’s main symptoms and the risk-benefit assessment of starting treatment. The importance of cognitive-behavioral treatment is also highlighted.

**Conclusions:**

Depression is one of the most common psychiatric disorders seen in PD. Depressive symptoms in PD are associated with increased motor disability and decreased quality of life. It is estimated that up to 50% of patients have depressive symptoms.

In conclusion, the approach to depression in Parkinson’s Disease must be multidisciplinary and comprehensive, with both psychopharmacological and psychotherapeutic treatment.

**Disclosure of Interest:**

None Declared

